# Comparative Molecular Effects of Dexmedetomidine and Propofol on Osteoblast Migration and Osteogenic Gene Expression at Pediatric-Equivalent Concentrations: An In Vitro Study

**DOI:** 10.3390/cimb48040392

**Published:** 2026-04-10

**Authors:** İlhan Kaya, Günseli Çubukçuoğlu Deniz, Merve Hayriye Kocaoğlu, Duru Aras Tosun, Akif Demirel

**Affiliations:** 1 Department of Oral and Maxillofacial Surgery, Faculty of Dentistry, Bursa Uludağ University, 16120 Bursa, Turkey; 2 Stem Cell Institute, Ankara University, 06520 Ankara, Turkey; gdeniz@ankara.edu.tr; 3 Department of Pediatric Dentistry, Faculty of Dentistry, Ankara University, 06560 Ankara, Turkey; mhkocaoglu@ankara.edu.tr (M.H.K.); akifdemirel@ankara.edu.tr (A.D.); 4 Department of Basic Medical Sciences, Faculty of Dentistry, Ankara University, 06560 Ankara, Turkey; datosun@ankara.edu.tr

**Keywords:** dexmedetomidine, jawbone healing, pediatric anesthesia, propofol, osteoblasts

## Abstract

This study compared the wound-healing response and osteogenic gene expression profile of osteoblasts exposed to pediatric-equivalent concentrations of dexmedetomidine (DXMT) and propofol (POF). Human osteoblast-like SAOS-2 cells were assigned to control, low- and high-dose DXMT and POF groups based on pharmacokinetically derived free-drug levels. Scratch-wound closure was quantified over 24 h, and expression of osteogenesis- and cytoskeleton-related genes (RANKL, RUNX2, SP7, BMP2, VIM, VCL, OCN, ALP) was measured by SYBR Green quantitative Polymerase Chain Reaction (qPCR). Normality was assessed using the Shapiro–Wilk test, and group differences were analyzed with two-way ANOVA followed by Tukey’s multiple comparisons test (*p* < 0.05). All groups demonstrated complete scratch closure by 24 h, with no differences at 6 h. At 18 h, POF did not differ from the control, whereas DXMT significantly accelerated closure at both doses in a dose-dependent fashion. High-dose DXMT significantly increased VIM (3.95 ± 3.12, *p* = 0.0144) and BMP2 (2.28 ± 0.70, *p* = 0.0002) expression, while RUNX2, SP7, and RANKL remained comparable to controls. ALP (1.68 ± 0.40, *p* = 0.0005) and OCN (3.31 ± 0.35, *p* = 0.0108) were significantly elevated only in the high-dose DXMT group, whereas POF showed no significant effects. At clinically relevant concentrations, DXMT was associated with enhanced scratch closure and increased expression of selected osteogenesis- and cytoskeleton-related genes in SAOS-2 cells, whereas POF showed limited effects under the tested conditions. These findings suggest that DXMT may influence early in vitro cellular responses relevant to bone healing and should be further validated in functional differentiation models and in vivo studies.

## 1. Introduction

Advanced caries, untreated endodontic infections, apical periodontitis, traumatic dentoalveolar injuries and osteomyelitis can lead to inflammatory lesions in the jawbones and loss of mineralized tissue [1,2,3,4,5,6,7,8,9,10]. Furthermore, traumatic injuries can trigger pulpal and periapical damage in primary and young permanent teeth, leading to long-term bone sequelae and remodeling responses. Therefore, early diagnosis, regular radiographic monitoring and appropriate treatment algorithms are critical in this group [8,9].

Numerous studies have demonstrated that periapical/alveolar bone lesions can show improvement over time following appropriate endodontic treatment, regenerative therapy or surgical approaches [11,12,13,14,15]. Regenerative endodontic treatments and current protocols can positively influence periapical bone healing and microarchitecture, especially in immature permanent teeth; however, the optimization of experimental strategies to support biological healing in the pediatric patient group remains an area of ongoing research [16,17,18,19].

Growing evidence suggests that agents commonly used for sedation and anesthesia in pediatric dental patients, such as dexmedetomidine (DXMT) and propofol (POF), may influence bone biology beyond their primary anesthetic effects. It has been reported that DXMT protects osteoblasts against oxidative stress, reduces apoptosis and may increase osteoblast activity; it may also support bone healing in experimental models [20,21]. On the other hand, the literature on POF is heterogeneous: increased osteoblast proliferation and osteogenic markers under hypoxia/reoxygenation conditions have been reported, and it has been shown to suppress Receptor Activator of Nuclear Factor-κB Ligand (RANKL)-mediated pathways on osteoclastogenesis. However, some studies have also reported that it reduces odontogenic/osteogenic differentiation. These conflicting findings point to the influence of different cell lines, stress conditions, and concentrations [22,23,24].

Studies directly comparing the osteogenic effects of these agents on osteoblasts at clinically relevant in vitro concentrations, particularly in the context of pediatric dentistry, remain limited. Addressing this gap may improve understanding of the biological safety and potential bone-healing relevance of sedation/anesthesia protocols. Therefore, this study aimed to compare the early in vitro effects of pediatric-equivalent concentrations of DXMT and POF on scratch-wound closure and the expression of osteogenesis-related genes in SAOS-2 cells. To this end, an in vitro scratch assay was used as a surrogate model of early wound-closure dynamics, and a panel of osteogenesis-related genes was analyzed to characterize early transcriptional responses, including the osteoblast differentiation factors RUNX2 and SP7/Osterix (SP7) [25], the osteoinductive mediator BMP2 [26], the bone remodeling marker RANKL [27], the migration-related markers VIM [28,29] and VCL [30,31], and the maturation/mineralization markers OCN/BGLAP [32] and ALP [33].

The null hypothesis (H0) of this study was that there would be no statistically significant differences between DXMT and POF regarding their effects on osteoblast wound healing and osteogenic gene expression at pediatric-equivalent concentrations. Accordingly, the subsequent sections describe the experimental workflow and analytical methods used to evaluate these outcomes.

## 2. Materials and Methods

### 2.1. Experimental Framework

Human bone osteosarcoma cells (SAOS-2 An_1_, catalogue no: 02111901) were obtained from the ŞAP Institute Laboratory (Ministry of Agriculture and Forestry, Ankara, Republic of Türkiye) and cultured under standard conditions at 37 °C and 5% CO_2_. Unless stated otherwise, all reagents were obtained from Sigma-Aldrich (St. Louis, MO, USA). The cultures were allocated into one control and four treatment groups; the control group received no pharmacological agent. For the experimental groups, clinically used lowest and highest pediatric doses of DXMT (0.5–1 µg/kg/h) [34] and POF (1–3 mg/kg/h) [35,36,37,38] were utilized as the basis for concentration selection. To bridge the gap between in vivo dosing and in vitro exposure, the tested concentrations were derived by accounting for differences in protein binding between human plasma and cell culture media. In pediatric patients, DXMT plasma concentrations typically range from 0.5 to 1.2 ng/mL [39,40], while POF maintenance sedation targets concentrations of 2.0–3.0 µg/mL [36]. In human plasma, POF is 98–99% protein-bound (primarily to albumin and erythrocytes), leaving a free fraction (fu) of only 1.2–1.7% [41]. However, in cell culture media supplemented with 10% Fetal Bovine Serum (FBS), the lower total protein content increases the unbound fraction of lipophilic drugs significantly [42].

Target free-drug levels derived from pediatric pharmacokinetic data were converted into nominal culture concentrations using the following general equation:

C*_nominal_* = C*_free_*/*f_u_*

where C*_free_* is the desired unbound drug level and *f_u_* is the unbound fraction in 10% FBS. Based on these calculations, DXMT was applied at 15 and 30 nM while POF was tested at 10 µM and 50 µM, representing the clinically achievable low and high exposure ranges for pediatric patients. These values represent the clinically achievable high-exposure range, accounting for the reduced protein buffering capacity of 10% FBS compared to whole blood [42].

Following drug exposure, all groups were evaluated using a standardized wound-healing (scratch) assay to assess cell migration and regenerative response. In parallel, osteogenic activity was quantified by quantitative polymerase chain reaction (qPCR) targeting key osteogenesis-related genes. All experiments were performed in triplicate under identical culture conditions to ensure reproducibility.

### 2.2. Cell Culture Workflow

**Figure 1:**
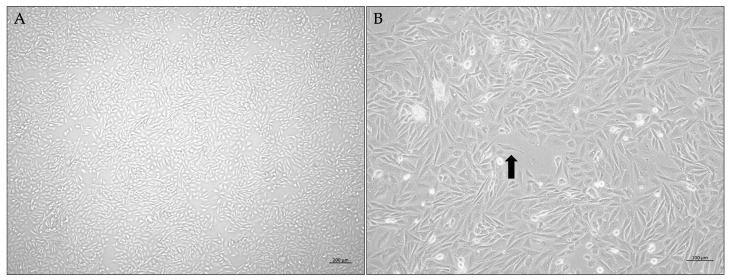
(**A**) At 4× magnification, osteoblast cultures show a fully confluent monolayer with uniform coverage across the field. (**B**) At 10× magnification, individual cells exhibit the characteristic spindle-shaped osteoblastic phenotype, highlighted by the arrow.

Osteoblast cells were cultured in RPMI-1640 medium supplemented with 10% fetal bovine serum, 1% penicillin–streptomycin, and 1% amphotericin. Cultures were established from cryopreserved stocks and maintained within a controlled passage range to minimize phenotypic variation. Osteoblast cells displayed a typical fibroblast-like phenotype, characterized by elongated spindle-shaped morphology (Figure 1). The medium was replaced every 2–3 days to ensure adequate nutrient supply, and cell morphology and confluency were routinely assessed by phase-contrast microscopy. Sub-culturing was conducted at 70–80% confluency using trypsin-Ethylene Diamine Tetra Acetic Acid (EDTA), and cells were seeded into 25 cm^2^ culture flasks or multi-well plates depending on the experimental design. All experiments were carried out with cells in the growth phase (*log* phase) to ensure consistency and reproducibility. The logarithmic growth phase for SAOS-2 cell line had been determined in our previous work [43].

### 2.3. Toxicity Assays

- Cell Viability Assay (WST-1)

To evaluate the potential cytotoxic effects of the anesthetics, a WST-1 ((4-[3-(4-Iodophenyl)-2-(4-nitro-phenyl)-2H-5-tetrazolio]-1,3-benzene sulfonate) colorimetric assay was performed. Each experimental condition was evaluated in three independent biological replicates.

SAOS-2 cells were seeded into 96-well plates at a density of 5 × 10^3^ cells/well and pre-cultured for 24 h to allow for attachment. The culture medium was then replaced with fresh medium containing the maximum clinical equivalent concentrations of the anesthetics: 30 nM DXMT and 50 µM POF. Cells incubated in a drug-free medium served as the control group. After 24 h of exposure, the medium was replaced with 100 µL of fresh medium containing 10 µL of the WST-1 reagent (Sigma-Aldrich). Following a 3-h incubation at 37 °C in the dark, the absorbance was measured at 450 nm (with a reference wavelength of 630 nm) using a microplate reader. Cell viability was expressed as a percentage relative to the untreated control group.

- Apoptosis Assessment (TUNEL Assay)

To definitively determine whether the anesthetics induce apoptotic cell death, a Terminal deoxynucleotidyl transferase dUTP nick end labeling (TUNEL) assay was conducted using a commercial kit according to the manufacturer’s instructions. Apoptosis analyses were performed in three independent biological replicates; five random microscopic fields were evaluated per replicate. Briefly, cells treated with 30 nM DXMT or 50 µM POF for 24 h were fixed with 4% paraformaldehyde and permeabilized with 0.1% Triton X-100. Cells were then incubated with the TUNEL reaction mixture. Nuclei were counterstained with 4′,6-diamidino-2-phenylindole (DAPI). The apoptotic index was calculated as the percentage of TUNEL-positive (green fluorescent) nuclei relative to the total number of DAPI-stained nuclei across five randomly selected fields of view under a fluorescence microscope.

- Cellular Proliferation Assay (Ki-67 Immunostaining)

The effect of the anesthetics on osteoblast proliferative capacity was assessed via Ki-67 immunofluorescence staining. Proliferation analyses were performed in three independent biological replicates; five independent microscopic fields were analyzed per replicate. Following 24 h of drug exposure (30 nM DXMT and 50 µM POF), the cells were fixed, permeabilized, and blocked. The cells were then incubated overnight at 4 °C with a primary antibody against Ki-67, followed by incubation with a fluorophore-conjugated secondary antibody for 1 h at room temperature. DAPI was used to stain all cell nuclei. The proliferation index was determined by calculating the percentage of Ki-67-positive nuclei out of the total nuclei counted in five independent microscopic fields.

### 2.4. Dynamic Wound-Healing Assay

For establishing the in vitro wound-healing model, equal numbers of SAOS-2 cells were seeded into 6-well plates and grown until they reached confluency. Once a fully confluent monolayer was achieved, a straight scratch was created using a sterile 200-µL pipette tip held perpendicular to the plate. The tip was gently drawn across the cell surface to produce a consistent wound gap. Detached cells and debris were then removed by washing with pre-warmed Phosphate-Buffered Saline (PBS), and fresh culture medium was added before imaging and further incubation.

To ensure consistent imaging, the borders of the “field of view” were marked with a fine-tip acetate marker as reference points. Images were captured at 4× magnification at 0, 6, 12, 18, and 24 h to monitor osteoblast migration toward the wound area. Wound closure was quantified in Fiji (ImageJ 1.53, National Institutes of Health, Bethesda, MD, USA) by applying threshold-based segmentation on calibrated 4× images to measure the remaining wound area at each time-point, consistent with methodologies previously described for evaluating propofol-mediated cell migration [44]. The system was calibrated to convert pixel measurements into square micrometers (µm^2^). The remaining wound area (expressed in µm^2^) at the specific time points was calculated and subsequently compared across the experimental groups to evaluate migration dynamics.

### 2.5. Quantitative Gene Expression Profiling

#### 2.5.1. RNA Isolation and cDNA Synthesis

RNA isolation and cDNA synthesis were performed following the procedures described in a previous study [43], with minor adaptations. Each condition was analyzed in three independent biological replicates, with three technical replicates for each biological replicate. Briefly, total RNA was extracted from SAOS-2 cells using TRIzol Reagent (Thermo Fisher Scientific, Waltham, MA, USA), and purified RNA was dissolved in nuclease-free water after ethanol washing. RNA concentration and purity were verified spectrophotometrically, and only samples meeting quality thresholds were used for downstream analysis. Subsequently, 1 µg of total RNA was reverse transcribed into cDNA using the Transcriptor First Strand cDNA Synthesis Kit (Roche Diagnostics GmbH, Mannheim, Germany) with random hexamers.

#### 2.5.2. qPCR

To assess drug-induced alterations in osteogenic activity, the expression of key genes representing different stages of osteoblast differentiation and bone remodeling was quantified following 24 h of cell culture with or without drug exposure. These included RANKL, RUNX2, SP7/Osterix, BMP2, VIM, VCL, OCN, and ALP, selected for their roles in osteoclast regulation, lineage commitment, osteoinduction, cytoskeletal organization, matrix mineralization, and late osteoblast maturation. Primer sequences specific to this study are provided in Supplementary Table S1, as they differ from those used in our previous work.

qPCR was performed using the LightCycler^®^ 480 SYBR Green I Master Mix on the LightCycler^®^ 480 System (Roche Diagnostics GmbH, Mannheim, Germany). Each condition was analyzed with three biological and three technical replicates. Specificity was confirmed by melting-curve analysis. Relative gene expression was calculated by the 2∽ΔΔCt method, normalized to glyceraldehyde-3-phosphate dehydrogenase (GAPDH) [45].

### 2.6. Statistical Analysis

Statistical analyses were performed using GraphPad Prism 10 (GraphPad Software, Boston, MA, USA). Data distribution was assessed using the Shapiro–Wilk normality test. Given the multifactorial design of the study, Two-Way Analysis of Variance (ANOVA) was employed for both assays to provide a robust assessment of interactions. Following ANOVA, multiple comparisons were performed using Tukey’s multiple comparisons test to identify significant differences between all study groups. Results were expressed as mean ± standard deviation (SD), and a *p*-value of <0.05 was considered statistically significant.

## 3. Results

### 3.1. Comparative Evaluation of Cell Migration Dynamics

**Figure 2:**
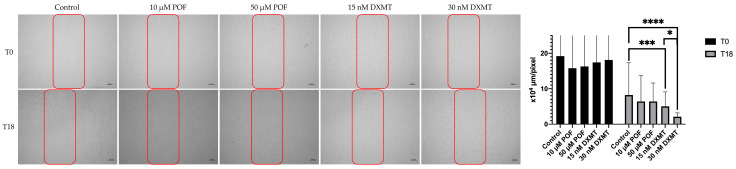
Representative scratch assay images of control, POF (low and high dose), and DXMT (low and high dose) groups acquired at t_0_ and t_18_. Regions of interest (ROIs) are indicated by red bounding boxes. By 24 h, scratch areas were fully closed in all groups; therefore, quantitative analyses were performed using the interval between t_0_ and t_18_. At t_18_, both low- and high-dose DXMT groups showed a dose-dependent increase in wound closure, exhibiting statistically significant improvement compared with their respective control groups [Five error bars at t_0_ were clipped at the axis limit to improve readability of the graph [(* *p* < 0.05, *** *p* < 0.001 **** *p* < 0.0001)].

In this study, wound-healing dynamics were evaluated with a scratch assay by comparing low- and high-dose DXMT groups (15 and 30 nM) and low- and high-dose POF groups (10 and 50 µM) with the untreated control at four time points: 0, 6, 18, and 24 h. Microscopic images obtained at 24 h demonstrated complete scratch closure across all groups, consequently precluding quantitative analysis at this final time point. At 6 h, none of the DXMT or POF concentrations produced a statistically significant deviation from the control, indicating that early migratory activity had not yet diverged among the treatment groups. By 18 h, the effects of the two agents began to diverge: while both POF doses exhibited a closure pattern comparable to that of the control with no statistically meaningful separation (8.2 ± 0.9, 7.5 ± 1.2 and 6.0 ± 0.7 × 10^4^ µm/pixel; *p* = 0.8930 and *p* = 0.0751 for control, 10- and 50 µM POF, respectively), DXMT demonstrated a clear and measurable enhancement in wound-healing capacity at both concentrations tested (4.4 ± 0.5 and 1.7 ± 0.5 × 10^4^ µm/pixel; *p* = 0.0008 and *p* < 0.0001 for 15- and 30 nM DXMT, respectively) (Figure 2). Moreover, the magnitude of this improvement displayed a dose-dependent trend, with the higher DXMT dose producing a more pronounced reduction in wound area (*p* = 0.0163). Although POF also showed a numerical, dose-related tendency toward accelerated closure, this change did not reach statistical significance (*p* = 0.3548) (Figure 2). Together, these findings indicate that, under the present experimental conditions, DXMT exerts a more robust and dose-responsive promotive effect on osteoblast migration than POF, which remained largely indistinguishable from the untreated control.

### 3.2. Toxicity Assays

- Cell Viability Assay (WST-1)

**Figure 3:**
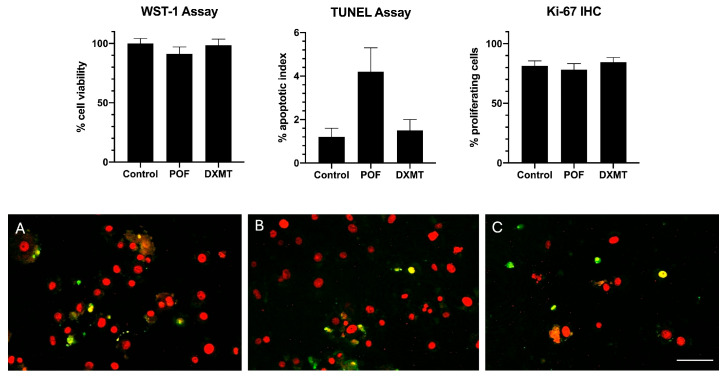
Toxicity assay results. Evaluation of osteoblast apoptosis following anesthetic exposure via TUNEL assay. Representative fluorescence microscopy images of SAOS-2 cells incubated for 24 h. (**A**) Untreated control group. (**B**) High-dose Dexmedetomidine (DXMT, 30 nM) group. (**C**) High-dose Propofol (POF, 50 µM) group. Total cell nuclei are counterstained in red, while apoptotic nuclei with fragmented DNA are stained in green (TUNEL-positive). Colocalized signals appear yellow. The scarcity of apoptotic cells across all panels visually confirms that neither DXMT nor POF induces significant cytotoxicity or cell death at the tested maximum pediatric-equivalent concentrations. (Scale bar = 100 µm).

To assess the potential cytotoxicity of the anesthetics at their maximum tested clinical equivalent concentrations, a WST-1 cell viability assay was conducted comparing the untreated control to the high-dose groups: 30 nM DXMT and 50 µM POF. The quantitative analysis of cell viability revealed no statistically significant differences among the groups (*p* > 0.05). The mean relative cell viability was 100.0 ± 4.2% for the control group, 98.5 ± 5.1% for the high-dose DXMT group, and 91.2 ± 5.8% for the high-dose POF group. These results confirm that neither DXMT nor POF induces significant cytotoxicity in SAOS-2 cells at the highest tested pediatric-equivalent concentrations (Figure 3).

- Cell Viability and Apoptosis Assessment (TUNEL Assay)

To assess the potential cytotoxicity of the anesthetics at their maximum tested clinical equivalent concentrations, a TUNEL assay was conducted comparing the untreated control to the high-dose groups: 30 nM DXMT and 50 µM POF. The quantitative analysis of TUNEL-positive (apoptotic) nuclei relative to the total cellular nuclei revealed no statistically significant differences among the groups (*p* > 0.05). The mean apoptotic index was 1.2 ± 0.4% for the control group (Figure 3A), 1.5 ± 0.5% for the high-dose DXMT group (Figure 3B), and 4.2 ± 1.1% for the high-dose POF group (Figure 3C). These results confirm that neither DXMT nor POF induces significant apoptosis in SAOS-2 cells at the highest tested pediatric-equivalent concentrations.

- Cellular Proliferation Assessment (Ki-67 Immunostaining)

To further evaluate the impact of the anesthetics on the proliferative capacity of osteoblasts, Ki-67 immunostaining was performed on the control and high-dose groups. The quantitative analysis revealed a robust baseline proliferative activity across all groups, consistent with the logarithmic growth phase of the cells. The percentage of Ki-67 positive nuclei (proliferation index) was 81.4 ± 4.2% in the untreated control group. In the high-dose DXMT (30 nM) and high-dose POF (50 µM) groups, the mean Ki-67 positivity was 84.6 ± 3.8% and 78.2 ± 5.1%, respectively. Statistical analysis indicated no significant differences among the groups (*p* > 0.05). These results demonstrate that neither DXMT nor POF suppresses the proliferative potential of osteoblasts at the tested maximum pediatric-equivalent concentrations. The robust preservation of the proliferation machinery across all groups confirms that the accelerated wound closure observed specifically in the DXMT group is synergistically driven by active cellular migration rather than merely an artifact of differential proliferation (Figure 3).

### 3.3. Gene Expression Analysis

#### 3.3.1. Comparative Profiling of Transcription Factors Involved in Osteoblast Differentiation

**Figure 4:**
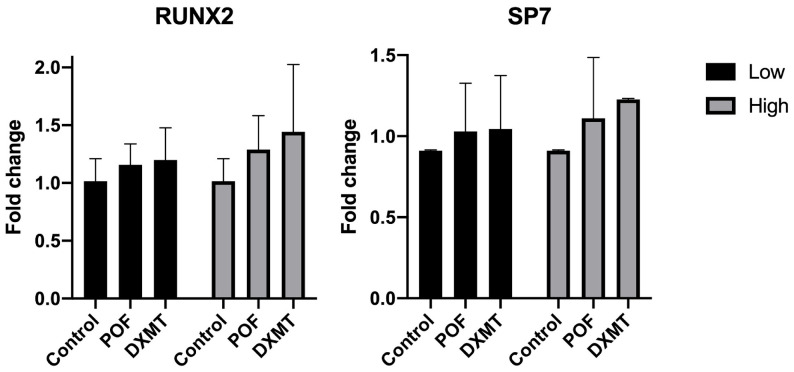
Expression levels of RUNX2 and SP7 after low- and high-dose POF and DXMT exposure. No significant differences were observed across groups, although both agents showed a mild dose-related upward trend.

For the early osteoblast transcription factors RUNX2 and SP7, quantitative analysis revealed no statistically significant differences between any of the treatment groups and the control. In both genes, expression levels in the POF groups (RUNX2: 1.16 ± 0.18 and 1.29 ± 0.30 fold, *p* = 0.7808 and *p* = 0.4148; SP7: 1.03 ± 0.30 and 1.11 ± 0.37 fold, *p* = 0.9063 and *p* = 0.7573 for 10 and 50 µM, respectively) and the DXMT groups (RUNX2: 1.20 ± 0.28 and 1.44 ± 0.58 fold, *p* = 0.6636 and *p* = 0.1383 fold; SP7: 1.04 ± 0.33 and 1.23 ± 0.01 fold, *p* = 0.8293 and *p* = 0.5148 for 10 and 50 µM, respectively) showed numerically higher values compared with the control (1.02 ± 0.19 and 0.91 ± 0.03 fold for RUNX2 and SP7, respectively); however, these changes did not reach statistical significance (Figure 4). Overall, RUNX2 and SP7 expression remained within a comparable range to the control across all treatment groups.

#### 3.3.2. Analysis of Cell Migration and Adhesion-Related Gene Markers

**Figure 5:**
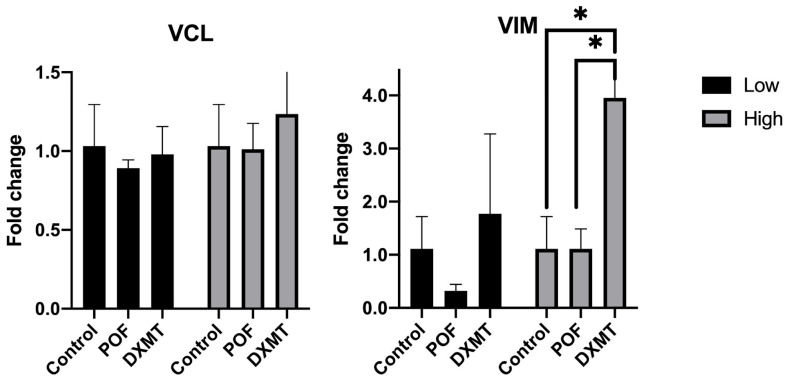
Expression levels of VCL and VIM after POF and DXMT exposure. VCL showed no significant changes, with slight decreases in POF and mild increases in DXMT groups. VIM was significantly elevated only in the high-dose DXMT group [Some of the error bars at t_0_ were clipped at the axis limit to improve readability of the graph (* *p* < 0.05)].

For VCL, no statistically significant differences were detected among the treatment groups. A reduction was observed in the low-dose POF and DXMT condition (10 µM POF: 0.89 ± 0.05 and 15 nM DXMT: 0.98 ± 0.18 fold) compared to the controls (1.03 ± 0.26 fold), and a similar decrease was noted at the high POF dose (50 µM POF: 1.01 ± 0.16), although neither reached statistical significance (*p* = 0.7959, *p* = 0.9686, and *p* = 0.9957, respectively) (Figure 5). High dose DXMT showed an increase in VCL expression (1.24 ± 0.46 fold), but these changes also did not achieve statistical significance (*p* = 0.6188) (Figure 5). Overall, VCL levels remained broadly comparable to the control across all tested conditions.

For VIM, high-dose DXMT produced a statistically significant increase in expression relative to both the control and POF groups (control: 1.11 ± 0.61; 10 µM POF: 0.32 ± 0.12; 50 µM POF: 1.11 ± 0.37; 30 nM DXMT: 3.95 ± 3.12 fold; *p* = 0.0144 for control vs. high-dose DXMT; *p* = 0.0451 for high-dose POF vs. DXMT). Low-dose DXMT likewise exhibited an upward trend (15 nM DXMT: 1.77 ± 1.50), consistent with dose-responsiveness, though this did not reach statistical significance (*p* = 0.7338) (Figure 5).

#### 3.3.3. Comparative Assessment of Osteoinduction-Associated Gene Expression

**Figure 6:**
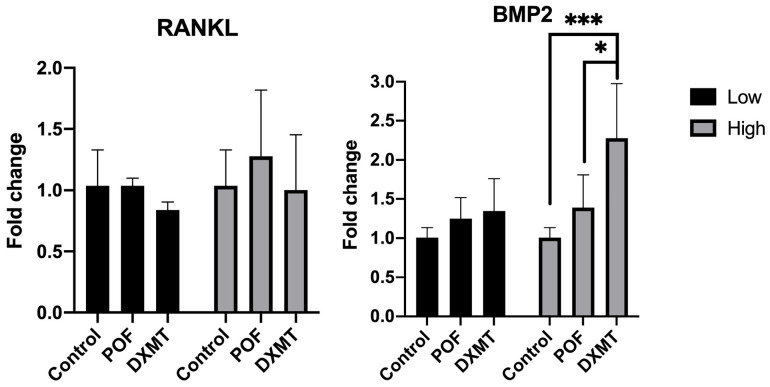
RANKL and BMP2 expression following POF (10–50 µM) and DXMT (15–30 nM) exposure. RANKL showed no significant differences among groups, with a slight increase at high-dose POF and dose-related decreases with DXMT. BMP2 exhibited a significant increase only in the high-dose DXMT group, while all other doses showed a non-significant upward trend [(* *p* < 0.05, *** *p* < 0.001)].

For RANKL, no statistically significant differences were detected among any of the treatment groups. Expression levels in the low-dose POF group were comparable to the control (control: 1.04 ± 0.29; 10 µM POF: 1.04 ± 0.06 fold; *p* > 0.9999), whereas the high-dose POF condition showed a numerical increase that did not reach statistical significance (50 µM POF: 1.28 ± 0.54 fold; *p* = 0.6209) (Figure 6). In contrast, both DXMT doses demonstrated a reduction in RANKL expression (15 nM DXMT: 0.84 ± 0.07; 30 nM DXMT: 1.00 ± 0.45 fold), although neither decrease was statistically significant (*p* = 0.7161 and *p* = 0.9895) (Figure 6). Overall, RANKL levels remained within a similar range to the control, despite these dose-related directional shifts.

For BMP2, a significant increase was observed only in the high-dose DXMT group, which differed from both the control and POF groups (control: 1.01 ± 0.14; 10 µM POF: 1.25 ± 0.27; 50 µM POF: 1.39 ± 0.42; 30 nM DXMT: 2.28 ± 0.70 fold; *p* = 0.0002 for control vs. high-dose DXMT; *p* = 0.0163 for POF vs. high-dose DXMT). Although both POF doses (10 and 50 µM) and the low-dose DXMT condition (15 nM) exhibited a dose-related upward trend relative to the control (15 nM DXMT: 1.35 ± 0.42 fold; *p* = 0.3178), these increases did not reach statistical significance (Figure 6). Overall, aside from the high-dose DXMT group, BMP2 expression remained statistically comparable to the control despite the presence of a consistent dose-dependent rise across both agents.

#### 3.3.4. Evaluation of Osteoblast Maturation and Mineralization Markers

**Figure 7:**
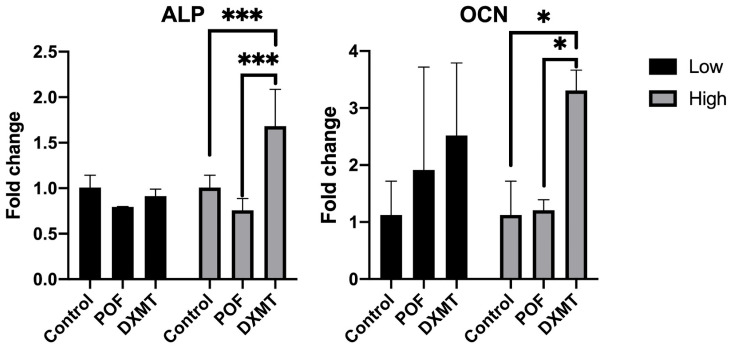
Expression levels of ALP and OCN following POF (10–50 µM) and DXMT (15–30 nM) exposure. Both markers showed significant increases only in the high-dose DXMT group, while POF produced no significant changes at either dose [(* *p* < 0.05, *** *p* < 0.001)].

For ALP, a significant increase was observed in the high-dose DXMT group compared with the control and POF conditions (control: 1.00 ± 0.13; 10 µM POF: 0.80 ± 0.01; 50 µM POF: 0.76 ± 0.13; 30 nM DXMT: 1.68 ± 0.40 fold, *p* = 0.0005 and *p* = 0.0002 for control and POF, respectively). Neither low-dose DXMT (15 nM: 0.91 ± 0.08 fold) nor any POF dose produced a statistically significant difference, despite the presence of a mild dose-related upward trend in all groups. Overall, ALP expression was elevated only under high-dose DXMT (Figure 7).

For OCN, expression similarly showed a significant increase exclusively in the high-dose DXMT group (30 nM DXMT: 3.31 ± 0.35 fold, *p* = 0.0108 and *p* = 0.0440 for control and POF, respectively), whereas both POF doses (10 and 50 µM: 1.91 ± 1.80 and 1.20 ± 0.18 fold) remained comparable to the control (control: 1.13 ± 0.59 fold). Low-dose DXMT (15 nM: 2.51 ± 1.27 fold) also failed to reach statistical significance (Figure 7). Thus, OCN expression was significantly enhanced only by high-dose DXMT, with no detectable effect of POF.

## 4. Discussion

The potential effects of sedative agents on tissue healing and bone remodeling, in addition to their effects on hemodynamic and behavioral responses, have attracted increasing attention in recent years. Dentoalveolar surgical procedures in pediatric dental patients are frequently performed under sedation or general anesthesia in individuals with comorbidities or limited cooperation. Therefore, it is imperative to comprehend the impact of agents incorporated within the anesthesia protocol on osteoblast function, inflammatory response, and the osseointegration process, as this is not only crucial for safety but also for ensuring long-term treatment success. DXMT, an α2-adrenergic agonist, has been reported to exhibit antioxidant, anti-inflammatory, and cell-protective properties in addition to its analgesic and sedative effects in various surgical and infection models [20,43,46]. Conversely, POF is frequently favored due to its hemodynamic stability and rapid recovery profile. Nevertheless, data concerning its effects on bone cells appear heterogeneous and somewhat contradictory [24]. Therefore, a direct comparison of the effects of these two commonly used sedatives on bone healing under the same experimental conditions is important to fill the existing knowledge gap. Although these findings may be relevant to early cellular responses associated with healing, they should not be used to guide clinical anesthesia protocols without further functional validation and in vivo evidence. Although the tested concentrations were selected on the basis of clinically used pediatric dosing ranges, they should not be interpreted as exact surrogates of in vivo pediatric plasma or tissue exposure. In vitro systems cannot fully replicate the pharmacokinetic complexity of clinical sedation, including protein binding, tissue distribution, metabolism, clearance, and age-dependent variability. Therefore, the present concentrations are best considered clinically informed experimental approximations of pediatric exposure rather than complete representations of in vivo pediatric pharmacokinetics. This limitation is particularly relevant for highly protein-bound agents such as propofol, for which differences in albumin content, erythrocyte binding, and free-drug fraction between plasma and culture media may substantially influence the effective cellular exposure.

From a molecular and anesthetic perspective, the present findings indicate that dexmedetomidine elicits an early pro-regenerative osteoblast response that extends beyond its conventional sedative role. Under pediatric-equivalent exposure conditions, DXMT accelerated scratch-wound closure and was associated with significant upregulation of VIM, BMP2, ALP, and OCN, whereas RUNX2, SP7, and RANKL remained largely unchanged. This pattern may reflect modulation of downstream osteogenesis- and cytoskeleton-related responses rather than substantial changes in early lineage-associated transcription factors; however, the present data do not support definitive mechanistic conclusions. In contrast, propofol showed no significant effect on wound closure or on the tested osteogenesis-related markers, supporting a comparatively neutral profile in this in vitro setting. Taken together, these data position DXMT as an anesthetic agent with a distinct molecular footprint on osteoblast behavior, characterized by promotion of cell migration and selective activation of osteogenesis-associated pathways, while POF appears to exert limited transcriptional influence under the same clinically relevant conditions.

The present findings indicate that DXMT accelerates wound healing, particularly at high doses, and simultaneously increases BMP2, VIM, ALP, and OCN expression. In contrast, POF exhibits a biologically neutral profile. These results led to the rejection of the null hypothesis. Specifically, DXMT significantly enhanced wound healing compared with both the control and POF groups. This finding is consistent with previous studies suggesting that DXMT exerts protective and regenerative effects on bone tissue and osteoblasts. Recent mechanistic studies clarify the pathways responsible for this effect. Lou et al. [20] demonstrated that DXMT prevents delayed bone healing by activating the phosphoinositide 3-kinase/protein kinase B (PI3K/Akt) signaling pathway, which in turn enhances nuclear factor erythroid 2-related factor 2 (Nrf2)-mediated antioxidant defense system. Similarly, Wang et al. [47] reported that DXMT promotes osteogenesis-angiogenesis coupling through the regulation of Vascular Endothelial Growth Factor A (VEGFA), further supporting its role in tissue regeneration. Our observation of increased VIM expression suggests that DXMT may activate these cytoskeletal and migratory pathways via α2-adrenergic receptor stimulation, driving the rapid wound closure observed in the scratch assay. A study conducted on normal human fetal osteoblast cells demonstrated that DXMT reduced oxidative stress-induced cell death and supported osteoblastic differentiation [48]. In a postmenopausal osteoporosis model, DXMT has been shown to positively affect bone mineral density and osteogenic differentiation by regulating markers involved in the osteogenesis-angiogenesis relationship [47]. A 2025 study utilizing an in vitro osteomyelitis model demonstrated that therapeutic doses of DXMT led to a reduction in inflammatory cytokines, an augmentation in osteogenic markers, and a promotion of bone healing in the context of S. aureus-infected osteocyte-like cells [43]. The findings on wound healing in the present study corroborate these data and suggest that DXMT may enhance the osteoblast-derived healing response even in the absence of infection.

In contrast, no statistically significant difference was found between the POF and control groups in terms of wound healing. The current literature reports conflicting results regarding the effects of POF on bone cells. Regarding cytotoxicity, concerns about the high concentration (50 µM) of POF were allayed by recent findings using Fluorescence Lifetime Imaging Microscopy (FLIM), which demonstrated that POF concentrations up to 100 µM induce metabolic shifts in cancer cells (e.g., MDA-MB-231) without compromising cell viability [49]. Similarly, our results showed complete wound closure in the 50 µM POF group, confirming that this concentration did not induce cell death or inhibit migration in SAOS-2 cells. It has been reported that POF is not cytotoxic at clinically comparable doses in hypoxic osteoblast models and that it supports osteoblast proliferation and mineralization by increasing BMP-2, transforming growth factor beta 1 (TGF-β1), type I collagen, and osteocalcin expression [23]. Conversely, a study on dental pulp stem cells reported that POF suppressed odontogenic/osteogenic differentiation and reduced alkaline phosphatase activity [22]. Additionally, it has been shown to inhibit osteoclastogenesis and reduce bone resorption by lowering the RANKL/osteoprotegerin (OPG) ratio in certain in vitro models [24]. In the updated gene-expression analysis, RANKL showed relative elevation in the propofol group, while all remaining osteogenic markers favored DXMT, reinforcing the interpretation that propofol remains largely inert in this context and may even weakly bias cells toward a catabolic rather than anabolic signaling profile [50]. High-dose DXMT -corresponding to the upper clinical limit of pediatric sedation—produced a coherent pro-osteogenic transcriptional profile, with significant upregulation of ALP, OCN, BMP2, and VIM, while these markers remained unchanged in the propofol-treated and control groups. The divergence between VIM and VCL, despite both being cytoskeleton-associated genes, provides important mechanistic insight. VIM, an intermediate filament protein, is rapidly induced during early cytoskeletal remodeling and cell motility [28,29]; its increase is fully consistent with enhanced osteoinductive signaling mediated by BMP2 [51], which was also significantly elevated in the high-dose DXMT group. Conversely, VCL, a hallmark of stable and mature focal adhesions [30,31], did not change, indicating that DXMT does not promote a stabilized adhesion phenotype but rather favors a dynamic, migratory cytoskeletal configuration. On the contrary, the biological neutrality of POF observed in our study may be explained by its differential impact on angiogenic and osteogenic signaling compared to DXMT. While DXMT appears to upregulate regenerative factors, recent evidence by Yang et al. [44] indicates that POF may attenuate angiogenesis by suppressing VEGFA transcription via the protein kinase R-like endoplasmic reticulum kinase (PERK)/eukaryotic initiation factor 2 alpha (eIF2α)/transcription factor AP-2 gamma (TFAP2C) signaling axis. Furthermore, Choi et al. [22]. noted that POF could attenuate odontogenic differentiation in dental pulp stem cells. Therefore, unlike DXMT, which actively engages migratory and osteoinductive pathways (BMP2, VIM), POF appears to lack specific receptor-mediated osteostimulatory activity at clinical concentrations, resulting in a neutral wound-healing profile.

The absence of significant changes in RUNX2 and SP7, despite the increase observed in selected downstream markers such as BMP2, ALP, and OCN, may suggest that DXMT did not markedly alter early lineage-associated transcription factors within the single early time point examined in this study. Alternatively, these findings may reflect selective modulation of downstream osteogenesis-related responses under the tested conditions. A similar dissociation between early transcription factors and downstream osteogenic markers has also been reported previously [52]. This interpretation should be made cautiously, however, because SAOS-2 cells already exhibit an osteoblast-like phenotype, and the present design did not include sequential time-point analysis or long-term functional differentiation assays.

Addressing the safety profile of the tested anesthetics, our TUNEL assay results demonstrated that neither DXMT nor POF induced significant apoptosis at their highest clinical equivalent doses (30 nM and 50 µM, respectively). This confirms that the differences observed in the wound-healing assay were driven by cellular dynamics rather than differential cell death. However, an interesting, albeit statistically insignificant, trend was observed: the apoptotic rate in the high-dose POF group was slightly elevated compared to both the control and DXMT groups. While this mild upward trend does not cross the threshold of cytotoxicity, it might represent a subtle cellular stress response that partially contributes to the relatively slower wound-healing trajectory observed in POF-treated osteoblasts. In contrast, high-dose DXMT maintained a baseline level of cell viability perfectly mirroring the control. This pristine preservation of cellular health, combined with the active transcriptional upregulation of migratory (*VIM*) [53] and osteoinductive (*BMP2*) [54] genes, further explains the robust regenerative response of DXMT-treated osteoblasts. By preserving cell viability while enhancing the expression of migration- and osteogenesis-related markers, DXMT-treated cells may be better positioned to support the early phase of wound healing.

Several limitations of this study should be acknowledged. First, the mechanistic interpretation proposed here—namely that DXMT enhances osteoblast migration via cytoskeletal remodeling and migratory signaling—remains speculative, as we did not directly evaluate changes in cytoskeletal architecture or migration-related pathways using protein-level assays. Second, this study utilized a short-term in vitro model that lacks systemic influences and clinically relevant stressors, such as inflammation or infection. Third, while SAOS-2 cells provide a stable and reproducible osteoblast-like model, they are derived from osteosarcoma and may exhibit altered metabolic profiles compared to primary human osteoblasts. Finally, the restriction to an early time point (24 h) limits the assessment to acute responses and precludes the evaluation of sustained osteogenic differentiation. Future studies incorporating longer observation periods, primary human osteoblasts, functional osteogenic assays (e.g., matrix mineralization), and in vivo models will be necessary to determine the long-term biological and clinical significance of these findings.

## 5. Conclusions

In this short-term in vitro study, DXMT was associated with faster scratch closure and higher expression of selected osteogenesis- and cytoskeleton-related genes in SAOS-2 cells, whereas POF showed no significant changes under the tested conditions. These findings support distinct early cellular responses to the two agents; however, they do not establish sustained osteogenic differentiation or definitive bone-healing outcomes. Functional differentiation assays and in vivo studies are needed to determine the biological and clinical relevance of these observations.

## Data Availability

The datasets used and/or analysed during the current study are available from the corresponding author on reasonable request.

## Supplementary Materials

The following supporting information can be downloaded at: https://www.mdpi.com/article/10.3390/cimb48040392/s1.

## Abbreviations

The following abbreviations are used in this manuscript:

DXMT	Dexmedetomidine
POF	Propofol
RANKL	Receptor Activator of Nuclear Factor-κB Ligand
RUNX2	Runt-related Transcription Factor 2
SP7	Sp7/Osterix
BMP2	Bone Morphogenetic Protein 2
VIM	Vimentin
VCL	Vinculin
OCN	Osteocalcin
GLA	Gamma-carboxyglutamate
BGLAP	Bone gamma-carboxyglutamate protein
ALP	Alkaline Phosphatase
FBS	Fetal Bovine Serum
EDTA	Ethylene Diamine Tetra Acetic Acid
PBS	Phosphate-Buffered Saline
qPCR	Quantitative Polymerase Chain Reaction
DAPI	4′,6-diamidino-2-phenylindole
GAPDH	Glyceraldehyde-3-phosphate dehydrogenase
VEGFA	Vascular Endothelial Growth Factor A
FLIM	Fluorescence Lifetime Imaging Microscopy
PI3K	Phosphoinositide 3-kinase
Akt	Protein kinase B
Nrf2	Nuclear factor erythroid 2-related factor 2
TGF-β1	Transforming Growth Factor Beta 1
OPG	Osteoprotegerin
PERK	Protein kinase R-like Endoplasmic Reticulum Kinase
eIF2α	Eukaryotic Initiation Factor 2 Alpha
TFAP2C	Transcription Factor AP-2 Gamma
