# Supplementary material for: Comparative Molecular Effects of Dexmedetomidine and Propofol on Osteoblast Migration and Osteogenic Gene Expression at Pediatric-Equivalent Concentrations: An In Vitro Study
Source: Curr Issues Mol Biol. 2026 Apr 10;48(4):392. doi: 10.3390/cimb48040392 (PMC13114927; doi:10.3390/cimb48040392)
Supplement: Supplementary file 1 [file cimb-48-00392-s001.zip › cimb-4230946-supplementary.pdf]

Table 1. Gene names and primer sequences used in this study

Gene Symbol	Gene Name	Forward Primer Sequence	Reverse Primer Sequence
BMP2	Bone Morphogenetic Protein 2	5'-GTATCGCAGGCACTCAGGTCA-3'	5'-CCACTCGTTTCTGGTAGTTCTTC-3'
VCL	Vinculin	5'-TGAGCAAGCACAGCGGTGGATT-3'	5'-TCGGTCACACTTGGCGAGAAG-3'
RUNX2	RUNX Family Transcription Factor 2	5′-CGCAGTATGAGAGTAGGTGTCC-3′	5′-GGGTAAGACTGGTCATAGGACC-3′
SP7	Sp7 Transcription Factor	5′-TTCTGCGGCAAGAGGTTCACTC-3′	5′-GTGTTTGCTCAGGTGGTCGCTT-3′
ALPP	Alkaline Phosphatase, placental	5′-CAACGAGTCATCTCCGTGATG-3′	5′-ACCAGTTGCGGTTCACCGTGT-3′
OCN (BGLAP)	Bone Gamma-Carboxyglutamate Protein	5′-CGCTACCTGTATCAATGGCTGG-3′	5′-CTCCTGAAAGCCGATGTGGTCA-3′
RANKL (TNFSF11)	TNF Superfamily Member 11	5′-GCCTTTCAAGGAGCTGTGCAAAA-3′	5′-GAGCAAAAGGCTGAGCTTCAAGC-3′
VIM	Vimentin	5'-AGGCAAAGCAGGAGTCCACTGA-3'	5'-TTCTGGCGTTCCAGGGACTCAT-3'
GAPDH	Glyceraldehyde-3-Phosphate Dehydrogenase	5′- GTCTCCTCTGACTTCAACAGCG-3′	5′- ACCACCCTGTTGCTGTAGCCAA-3′
